# Spatio-Temporal Distribution Characteristics and Driving Factors of Main Grain Crop Water Productivity in the Yellow River Basin

**DOI:** 10.3390/plants12030580

**Published:** 2023-01-28

**Authors:** Yan Zhang, Feiyu Wang, Zhenjie Du, Ming Dou, Zhijie Liang, Yun Gao, Ping Li

**Affiliations:** 1 Farmland Irrigation Research Institute, Chinese Academy of Agricultural Sciences, Xinxiang 453002, China; 2 Key Laboratory of Water Cycle and Related Land Surface Processes, Institute of Geographic Sciences and Natural Resources Research, Chinese Academy of Sciences, Beijing 100101, China; 3 Laboratory of Quality and Safety Risk Assessment for Agro-Products on Water Environmental Factors, Ministry of Agriculture, Xinxiang 453002, China; 4 School of Ecology and Environment, Zhengzhou University, Zhengzhou 450001, China

**Keywords:** water productivity, discrepancy characteristics, driving factors, contribution rate, Yellow River Basin

## Abstract

To reveal the relationship between agricultural water resource consumption and grain production in the Yellow River Basin, the irrigation water productivity (WPI), crop water productivity (WPC), total inflow water productivity (WPT), and eleven influencing factors were selected. The spatial and temporal distribution characteristics and driving factors of water productivity of the main crops in the Yellow River Basin were analyzed with the spatial autocorrelation analysis, grey correlation analysis, sensitivity analysis, and relative contribution rate. The results showed that the minimum mean values of WPI, WPC, and WPT were 0.22, 0.35, and 0.18 kg/m^3^ in Qinghai, respectively, the maximum mean value of WPI was 2.11 kg/m^3^ in Henan, and the maximum mean values of WPC and WPT were 0.71 and 0.61 kg/m^3^ in Shandong, respectively. The changing trends in WPI and WPT in Qinghai and in WPC in Shandong were insignificant, whereas the WPI, WPC, and WPT in other provinces showed a significant increasing trend. Water productivity displayed a certain spatial clustering feature in the Yellow River Basin in different years, such as a high-high (H-H) aggregation in Henan in 2005, and an H-H aggregation in Shanxi in 2015 for WPI. The water productivity had a significant positive correlation with the consumption of chemical fertilizer with a 100% effective component (CFCEC), effective irrigated area (EIA), plastic film used for agriculture (PFUA), and total power of agricultural machinery (AMTP), while it had a significant negative correlation with the persons engaged in rural areas (PERA). There was a large grey correlation degree between the water productivity and the average annual precipitation (AAP), CFCEC, PFUA, consumption of chemical pesticides (CFC), and AMTP in the Yellow River Basin, but their sensitivity was relatively small. The main driving factors were EIA (8.98%), agricultural water (AW, 15.55%), AMTP (12.64%), CFCEC (12.06%), and CPC (9.77%) for WPI; AMTP (16.46%), CFCEC (13.25%), average annual evaporation (AAE, 12.94%), EIA (10.49%), and PERA (10.19%) for WPC; and EIA (14.26%), AMTP (13.38%), AAP (12.30%), CFCEC (10.49%), and PFUA (9.69%) for WPT in the Yellow River Basin. The results can provide support for improving the utilization efficiency of agricultural water resources, optimizing the allocation of water resources, and implementing high-quality agricultural developments in the Yellow River Basin.

## 1. Introduction

Highly efficient water use in agriculture is important for food security, water resource security, and ecological environment security [1]. The problem of water shortages has become increasingly prominent and has led to more intense competition among various water departments due to the rapid development of the social economy and the improvement in people’s living standards [2]. Farmland irrigation is seriously threatened and normal agricultural production cannot be guaranteed, which means that the limited agricultural water resources in the Yellow River Basin need to be utilized to produce more food [3]. Therefore, improving the utilization efficiency of agricultural water resources, alleviating the shortage of agricultural water, and improving the security of food supplies are of great significance for the high-quality development of the Yellow River Basin.

Water productivity is a comprehensive index used to measure the level of agricultural production and the scientificity and rationality of agricultural water use that can be divided into generalized, regional-scale water productivity and narrow-field, small-scale water productivity according to the different perspectives, scopes, and focuses of the analysis problem [4]. In order to improve field water productivity, relevant field management measures (high-efficiency irrigation systems, drought-resistant varieties, replacing water-intensive crops with less water-consuming crops, soil mulching, no-tillage systems, and other measures) have been proposed [5], and field water productivity has been mainly obtained through field-scale monitoring experiments [6,7,8]. For example, related studies have shown that introducing many water-saving irrigation strategies for rice (*Oryza sativa* L.) can improve its water productivity [9,10,11], such as through dry/wet alternate irrigation that dynamically regulates the dry and wet state of rice fields during rice growth. Dry/wet alternate irrigation has the potential to improve water productivity and rice yield compared with continuous flooding [12,13]. The main laws of water productivity of Xinjiang’s main grain crops under drip irrigation conditions were discovered using the plot experiment method [14]. A factorial field experiment using irrigation mode and plant population density was conducted in the west of Inner Mongolia to examine their effects on the water productivity of cotton (*Gossypium* spp.) [15]. Additionally, cotton water productivity was evaluated using different amounts of irrigation water and different drip irrigation techniques (surface drip irrigation and subsurface drip irrigation) [16]. Abebe et al. showed that irrigation depth had significant effects on the marketable yield and water productivity of broad beans (*Vicia faba* L.), whereas irrigation frequency had no significant effects on the marketable yield of broad beans [17].

For regional-scale water productivity, due to the influence of the uneven spatial and temporal distribution of populations, water resources, and climate conditions, water productivity also presents with an uneven distribution in space and time [18]. For example, Mainuddin and Kirby analyzed the water productivity of the main crops in the lower reaches of the Mekong River Basin and showed that water productivity has significant spatial variability, and the water productivity of various crops displays an increasing trend [19]. Yan and Wu found that the water productivity of winter wheat (*Triticum aestivum* L.) has significant spatial and temporal differences, and the water productivity steadily increases each year in the Haihe River Basin [20]. Li et al. pointed out that there is much room for improvement in irrigation water productivity in the Hexi Corridor of Gansu, China [21]. Kelley et al. analyzed the interannual variability of water productivity in the Snake Plains of eastern Idaho, USA. They showed that irrigated areas and crops with a sufficient water supply showed a general trend of an increase in production alongside an increase in water consumption, and the resulting water productivity roughly followed the linear function of actual evapotranspiration [22]. In addition, crop models, hydrological models, remote sensing, statistics, and geographic information systems are usually used to analyze regional-scale water productivity [23,24,25,26]. Gemechu et al. used remote sensing-derived datasets to map the seasonal and spatial variability of water productivity in sugarcane (*Saccharum officinarum* L.) agriculture in three large irrigation schemes (Wonji, Fincha’a, and Metahara) in Ethiopia [27]. Ahmadpour et al. proposed a joint estimation method of spatio-temporal variation of crop water productivity under a deficit irrigation scenario based on the AquaCrop model [28]. Flach et al. used the EPIC crop model to carry out a spatially explicit assessment of water consumption and water productivity under crop management scenarios in Brazil from 1990 to 2013 [29]. Liu et al. estimated the wheat yield and WP at a grid resolution of 30 arc minutes on the land surface by developing a GIS-based EPIC model [30]. Using remote sensing data products as input, Zwart et al. applied the WAT-PRO water productivity model at a global scale with global datasets of the NDVI and surface albedo to benchmark the water productivity of wheat at the beginning of this millennium [31].

In general, the uneven temporal and spatial distribution of water productivity is influenced by various factors. To analyze the temporal and spatial distribution characteristics and driving factors of major grain crop water productivity in the Yellow River Basin, we selected three water productivity indicators (i.e., irrigation water productivity, crop water productivity, and total inflow water productivity) and eleven influencing factors. The spatial and temporal differences of major grain crop water productivity, the response characteristics between water productivity and the influencing factors, and the relative contribution rate of each influencing factor to water productivity in the Yellow River Basin from 2000 to 2019 were comprehensively analyzed by using the spatial autocorrelation analysis, grey correlation analysis, sensitivity analysis, and relative contribution rate. The research results provide support for improving the utilization efficiency of agricultural water resources, optimizing water resource allocation, and implementing high-quality agricultural developments in the Yellow River Basin.

## 2. Materials and Methods

### 2.1. Study Area and Data Sources

**Figure 1:**
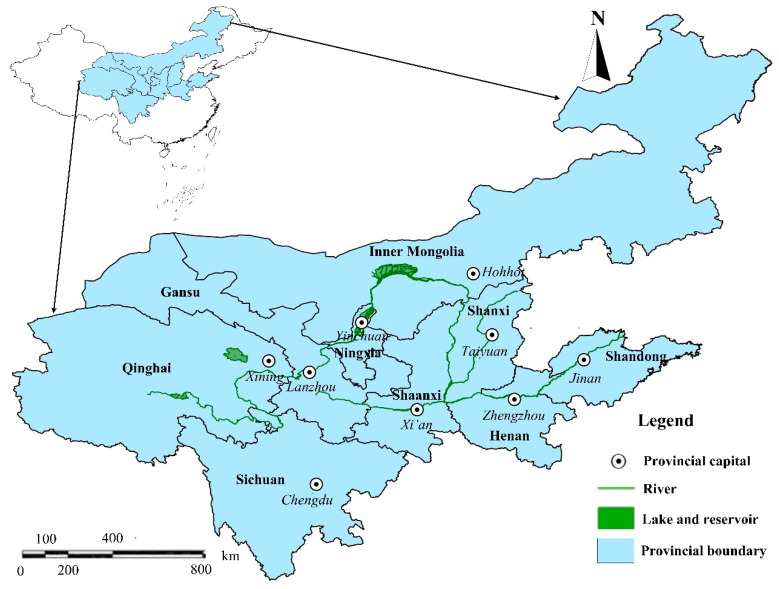
Distribution map of nine provinces (autonomous regions) in the Yellow River Basin.

The Yellow River is the second largest watershed in China, with a watershed area of 795,000 km^2^ that originates from the Qinghai–Tibet Plateau and has a total length of 5464 km. From west to east, the Yellow River flows through nine provinces (or autonomous regions), including Qinghai, Sichuan, Gansu, Ningxia, Inner Mongolia, Shaanxi, Shanxi, Henan, and Shandong, and finally empties into the Bohai Sea (Figure 1) [32,33]. As the main grain-producing area in China, the Yellow River Basin accounts for 36.03% of the country’s grain-sown area and 35.31% of the country’s grain output [3]. The annual average precipitation is about 466 mm, and the precipitation distribution decreases from southeast to northwest with large interannual changes in the Yellow River Basin [34]; the agricultural water consumption is 28.684 billion m^3^, accounting for 67.31% of the total water consumption in 2020. The Yellow River Basin has problems such as an increasingly prominent contradiction between water supply and demand, serious deterioration of water quality, and low utilization efficiency of agricultural water resources. Therefore, improving the utilization efficiency of agricultural water resources is an important method for guaranteeing the high-quality development of the Yellow River Basin.

The relevant research data were obtained from the China Rural Statistical Yearbook, China Water Resources Bulletin, and Statistical Yearbooks of the provinces (autonomous regions) from 2000 to 2019, and the meteorological data were obtained from the China Meteorological Data Network (Statistics from 1 January 2000 to 31 December 2019). In this study, eleven representative indicators (annual average precipitation, annual average evaporation, annual average temperature, agricultural water, consumption of chemical fertilizer with a 100% effective component, effective irrigated area, total sown area of grain crops (the grain crops mainly include wheat (*Triticum aestivum* L.) and corn (*Zea mays* L.)), plastic film used for agriculture, consumption of chemical pesticides, total power of agricultural machinery, and persons engaged in rural areas) were selected to evaluate the irrigation water productivity, crop water productivity, and total inflow water productivity of the provinces (autonomous regions) in the Yellow River Basin. The specific information is shown in Table 1.

### 2.2. Research Methods

#### 2.2.1. Water Productivity Index and Calculation Method

Water productivity refers to the yield or output value obtained per unit of water resources under certain crop varieties and cultivation conditions [35]. There are differences in the calculation methods of the water productivity index, and therefore, three water productivity indexes were used in this study. Irrigation water productivity (WPI) refers to the crop yield obtained per unit of irrigation water, which can comprehensively reflect the local irrigation engineering status and irrigation management level, and intuitively show the effect of irrigation water input on crop output. Crop water productivity (WPC) refers to the crop yield obtained per unit of water consumed. The total inflow water productivity (WPT) refers to the crop yield obtained per unit of total inflow water.

(1) Y=GCTO/GCTSA

(2) $$\mathrm{WPI}=Y/W_{t}$$

(3) WPC=Y/10ET

(4) $$\mathrm{WPT}=Y/(W_{t}+P_{t})$$

where *Y* is the per unit area yield, kg/hm^2^; GCTO is the total output of grain crops, kg; *W_t_* is irrigation water consumption per unit area, m^3^/hm^2^, which generally refers to the gross irrigation water consumption; *ET* is crop evapotranspiration, mm; and *P_t_* is annual precipitation, mm/hm^2^.

The crop evapotranspiration (*ET*) is calculated using the single-crop coefficient method, and the formula is as follows:

(5) $$ET=K_{c}ET_{0}$$

The Penman–Monteith formula recommended by the FAO is adopted to calculate the reference crop evapotranspiration (*ET*_0_), which can be expressed as:

(6) $$ET_{0}=\frac{0.408\Delta (R_{n}-G)+\gamma \frac{900}{T+273}u_{2}(e_{s}-e_{a})}{\Delta +\gamma (1+0.34u_{2})}$$

where *K_c_* is the comprehensive crop coefficient; *ET*_0_ is the reference crop evapotranspiration, mm; *G* is the soil heat flux, MJ/(m^2^·d); *R_n_* is the net radiation of the crop canopy, MJ/(m^2^·d); *T* is the average temperature at a height of 2 m, °C; *u*_2_ is the wind speed at a height of 2 m, m/s; *e_s_* is the saturation vapor pressure, kPa; *e_a_* is the actual vapor pressure, kPa; Δ is the slope of the saturation vapor pressure and temperature curve, kPa/°C; and γ is the wet and dry thermometer constant, kPa/°C.

#### 2.2.2. Mann–Kendall Trend Test

The Mann–Kendall (M–K) trend test is a nonparametric statistical test, which means it is a distribution-free test. The M–K trend test is not disturbed by a few outliers and is applicable to data testing of non-normal distribution hydrology, meteorology, and water-quality factor data [36]. The zero hypothesis (*H*_0_) of the M–K trend test is the time series *x_i_*_′_ (*i′* = 1, 2, …, *n*) and is an independent random variable with the same distribution of samples; the alternative hypothesis (*H*_1_) is a bilateral test that assumes that *x_i_*_′_ and *x_j_* (*j* ≤ *n* and *i′* ≠ *j*) have different distributions. The advantage of the M–K trend test is that it can test the linear or nonlinear variation trends, and its extended accuracy has been widely used in water quality trend analysis [37]. *Z_MK_* is the variation trend of time series data, which exhibits an increasing trend if *Z_MK_* is greater than zero and a decreasing trend if *Z_MK_* is less than zero. When |*Z_MK_*| > Z_(1−α/2)_, the null hypothesis is rejected, and there is a significant trend in the time series data. Values of Z_(1−α/2)_ can be found by using the standard normal distribution table; when the level of α = 5% is significant, the corresponding value is 1.96 [38].

#### 2.2.3. Spatial Autocorrelation Analysis

Spatial autocorrelation analysis [39,40,41] can reveal whether there is a correlation between data from a specific region and its adjacent regions. In this study, global spatial autocorrelation and local spatial autocorrelation analysis methods were used to identify the spatial agglomeration of water productivity levels in each province (or autonomous region) of the Yellow River Basin. Global spatial autocorrelation uses the Global Moran’s *I* coefficient to reflect the distribution effect of the unit in the study region. The calculation formula is as follows:

(7) $$I=\frac{n\sum_{i=1}^{n}\sum_{j=1}^{n}\left(X_{i}-\bar{X}\right)\left(X_{j}-\bar{X}\right)}{\sum_{i=1}^{n}{\left(X_{i}-\bar{X}\right)}^{2}\sum_{i=1}^{n}\sum_{j=1}^{n}W_{ij}}$$

where *n* is the number of spatial units in the study region, *X_i_* and *X_j_* are the observed values of region *i* and region *j*, and $\bar{X}$ is the average value of *X*. *W_ij_* is the spatial weight matrix, that is, the spatial proximity relationship between region *i* and *j*. If region *i* and *j* are adjacent, the weight is 1; otherwise, the weight is 0. *I*∈[−1, 1] when the significance level is given. If *I* > 0, it indicates that the water productivity level of the study region presents a spatial aggregation phenomenon. The larger the *I* value, the more obvious the spatial aggregation feature of the water productivity level and vice versa, which indicates that the water productivity level in the study region presents spatial differences.

After analyzing the global correlation, local autocorrelation is used to explore the spatial location and clustering situation of agglomeration centers to reflect the correlation degree of the same phenomenon or attribute value of local regional units in a large space. In this study, the LISA cluster map was obtained based on the local Moran’s I coefficient to explore the spatial aggregation degree of water productivity levels in each province (autonomous region) of the Yellow River Basin and their adjacent regions. The LISA aggregation types of local spatial autocorrelation can be divided into four types: high-high aggregation (H-H), high-low aggregation (H-L), low-high aggregation (L-H), and low-low aggregation (L-L).

#### 2.2.4. Grey Correlation Analysis

Grey correlation analysis is based on the grey system theory and is used to solve uncertainty problems, such as when there is incomplete information and fewer data. The basic idea is to study geometric features between indicators on the basis of mathematical methods. The more similar the geometric shapes are, the greater the degree of correlation is and vice versa [42,43]. In this study, the grey correlation degree method was used to calculate the correlation between the influencing factors and water productivity in each province (autonomous region) of the Yellow River Basin, and to analyze the response characteristics of water productivity changes. The main steps are as follows:

(8) $$\zeta_{0i}(k)=\frac{\Delta (\min)+\rho \Delta (\max)}{\Delta_{0i}\left(k\right)+\rho \Delta (\max)}$$

(9) $$r_{0i}=\frac{1}{N}\sum_{k=1}^{N}\zeta_{0i}(k)$$

where *ζ*_0*i*_(*k*) is the grey correlation coefficient of each comparison sequence in the *i*th year; Δ(min) and Δ(max) are the minimum and maximum absolute differences between each comparison sequence and the reference sequence when the indicator is dimensionless; *ρ* is the resolution coefficient, which is usually 0.5; Δ_0*i*_(*k*) is the absolute value difference between the comparison sequence and the reference sequence in the *i*th year when the indicator is dimensionless; *r*_0*i*_ is the average value of the correlation degree in the *i*th year of each comparison sequence of the indicator; and *N* is the number of moments in the reference sequence.

#### 2.2.5. Sensitivity Analysis

The sensitivity coefficient between water productivity and the influencing factors in each province (autonomous region) of the Yellow River Basin was calculated by using a sensitivity analysis, and the influencing factors with a strong sensitivity to the change in water productivity could be determined quantitatively. The specific calculation formula is [44,45]:

(10) $$\varepsilon =\frac{\bar{F}}{\bar{WP}}\cdot \frac{\sum \left(F_{i}-\bar{F})(WP_{i}-\bar{WP}\right)}{\sum (F_{i}-\bar{F}{)}^{2}}$$

where *ε* is the sensitivity coefficient of the influencing factor; *F_i_* is the *i*th value of the data series of the influencing factor; *WP_i_* is the *i*th value of the water productivity data series; and $\bar{F}$ and $\bar{WP}$ are the multi-year averages of the data series of influencing factors and water productivity, respectively. The water productivity increased with the increase in influencing factors when ε > 0, and the water productivity decreased with the increase in influencing factors when ε < 0; thus, the greater the absolute value of the sensitivity coefficient, the stronger the sensitivity.

#### 2.2.6. Relative Contribution Rate

The relative contribution rate was mainly used to quantitatively analyze the contribution rate of each influencing factor to the water productivity of each province (autonomous region) in the Yellow River Basin, and then was used to determine the dominant factor. Data standardization refers to the process of using transformation formula to process indicators with different attributes or orders of magnitude so that they can be compared and weighted. Therefore, due to the different dimensions and scopes of influencing factors and water productivity, it is necessary to standardize them first, and then analyze the relative contribution rate of each influencing factor to the change in water productivity via the multiple linear regression method [46,47].

(11) $$\overset{⌢}{WP}=k_{0}+k_{1}Z_{1}+k_{2}Z_{2}+k_{3}Z_{3}+\cdot \cdot \cdot +k_{n}Z_{n}$$

(12) $$\eta_{i}=\frac{\left|k_{i}\right|}{\left|k_{1}\right|+\left|k_{2}\right|+\left|k_{3}\right|+\cdot \cdot \cdot +\left|k_{n}\right|}(i=1,2,3,\cdots ,n)$$

where $\overset{⌢}{WP}$ is the standardized value of water productivity; *Z*_1_, *Z*_2_, *Z*_3_, …, *Z_n_* are the standardized values of the influencing factors; *k_i_* is the regression coefficient corresponding to the influencing factor; and *η_i_* is the relative contribution rate of the change in each influencing factor to the change in water productivity.

## 3. Results

### 3.1. Statistical Characteristics of Water Productivity in the Yellow River Basin

The statistical characteristics of water productivity in the Yellow River Basin are shown in Table 2. The mean values of WPI ranged from 0.22 to 2.11 kg/m^3^ in the Yellow River Basin and showed: Henan > Shandong > Shanxi > Sichuan > Inner Mongolia > Shaanxi > Gansu > Ningxia > Qinghai. The WPI showed weak variability in Sichuan and Shaanxi, where the variable coefficients were less than 10%, indicating that the variation amplitude of WPI was relatively small from 2000 to 2019. The WPI showed moderate variability in other provinces. The mean values of WPC ranged from 0.35 to 0.71 kg/m^3^ and showed: Shandong > Henan > Sichuan > Shanxi = Shaanxi > Ningxia > Inner Mongolia > Gansu > Qinghai. All provinces showed moderate variability for WPC. The mean values of WPT ranged from 0.18 to 0.61 kg/m^3^, and showed: Shandong > Henan > Inner Mongolia > Sichuan > Shanxi > Gansu > Shaanxi > Ningxia > Qinghai. The WPT showed weak variability in Qinghai, Sichuan, and Shaanxi, with variable coefficients of 9.15%, 5.83%, and 7.26%, respectively, indicating that the variation amplitude of WPT from 2000 to 2019 was relatively small, whereas it was moderate in other provinces.

In general, the water productivity displayed significant spatial differences in the Yellow River Basin that were roughly manifested by the relatively small/large level of water productivity in the western (such as Qinghai and Ningxia) and eastern (such as Shandong and Henan) regions, which was consistent with the distribution of water resources in the Yellow River Basin.

### 3.2. Spatial and Temporal Distribution of Water Productivity in the Yellow River Basin

#### 3.2.1. Temporal Variation Characteristics of Water Productivity

**Figure 2:**
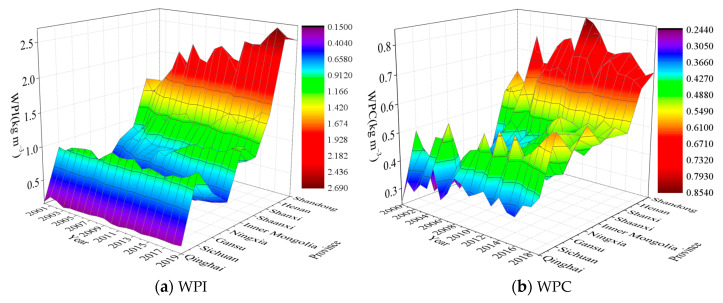

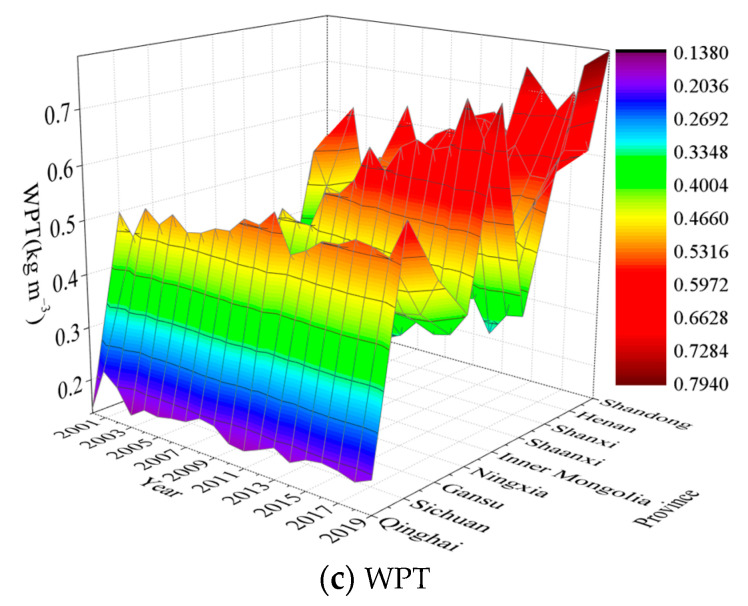
Temporal variation trend of water productivity in the Yellow River Basin: (**a**) the temporal variation trend of WPI, (**b**) the temporal variation trend of WPC, (**c**) the temporal variation trend of WPT.

The temporal variation trend of water productivity in the Yellow River Basin is shown in Figure 2. According to the M–K trend test, the changing trend of WPI in Qinghai was not significant, whereas the WPI in other provinces showed a significant increasing trend (Figure 2a). The WPI values of provinces (autonomous regions) was the lowest around 2001, as was seen, for example, when the WPI values of Ningxia, Shaanxi, and Shandong were the smallest at 0.15, 0.72, and 1.34 kg/m^3^ in 2000, 2001, and 2002, respectively; however, the maximum WPI values of Qinghai, Shanxi, and Shandong were 0.27, 1.54, and 2.48 kg/m^3^ in 2001, 2018, and 2019, respectively. Figure 2b indicates that the changing trend of WPC in Shandong was not significant, whereas the WPI in other provinces showed a significant increasing trend. The minimum WPC values in Qinghai, Shaanxi, Shandong, Henan, and Sichuan were 0.25, 0.34, 0.53, 0.50, and 0.40 kg/m^3^ in 2000, 2001, 2003, 2005, and 2006, respectively; however, the minimum WPC values in Shandong, Inner Mongolia, Shanxi, and Qinghai were 0.85, 0.52, 0.54, and 0.42 kg/m^3^ in 2010, 2015, 2016, and 2019, respectively. As shown in Figure 2c, the changing trend of WPT in Qinghai was not significant, whereas the WPI in other provinces showed a significant increasing trend. The minimum WPT values in Qinghai, Sichuan, and Shaanxi were 0.14, 0.46, and 0.25 kg/m^3^ in 2000, 2001, and 2002, respectively; however, the minimum WPT values in Qinghai, Shaanxi, and Sichuan were 0.22, 0.37, and 0.60 kg/m^3^ in 2001, 2016, and 2019, respectively.

#### 3.2.2. Spatial Differential Characteristics of Water Productivity

The Global Moran’s *I* coefficient of water productivity in the Yellow River Basin from 2000 to 2019 was calculated using the Euclidean space distance as the weight based on the Geoda software (Table 3). The Global Moran’s *I* coefficient of WPI, WPC, and WPT were all greater than 0, but failed to pass the significance test in the Yellow River Basin within the study time limit, and the Global Moran’s *I* coefficient showed that WPI > WPC > WPT, which indicates that the water productivity displayed a certain spatial clustering feature in the Yellow River Basin.

**Figure 3:**
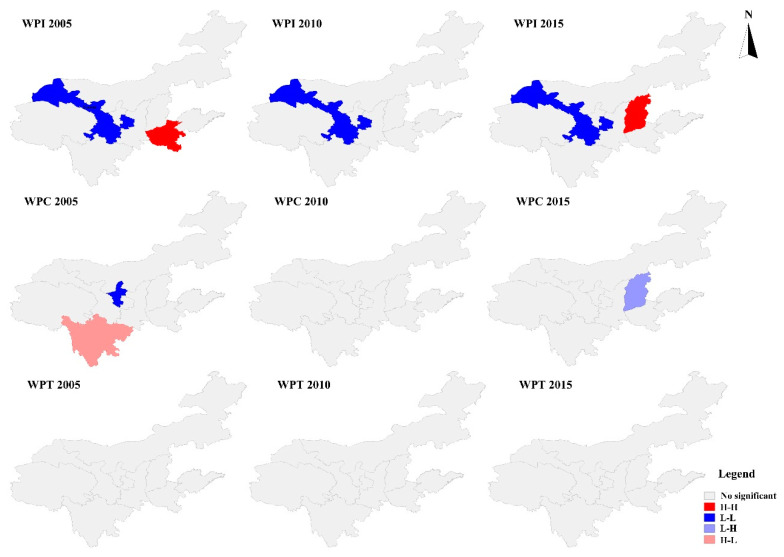
LISA cluster diagram of water productivity in the Yellow River Basin in 2005, 2010, and 2015.

The spatial agglomeration degree of the water productivity in the Yellow River Basin and its adjacent areas was analyzed in three typical years (2005, 2010, and 2015) (Figure 3). The WPI of Gansu showed L-L aggregation in 2005, 2010, and 2015; further, the WPI of Henan showed H-H aggregation in 2005, the WPI of Shanxi showed H-H aggregation in 2015, and the WPI of the other provinces showed no significant aggregation in 2005, 2010, and 2015. The WPC in Ningxia showed L-L aggregation in 2005, the WPC in Sichuan showed H-L aggregation in 2005, the WPC in Shanxi showed L-H aggregation in 2015, and the other provinces showed no significant aggregation in 2005, 2010, and 2015. The concentration of WPT in all provinces in 2005, 2010, and 2015 was not significant.

### 3.3. Response Characteristics between Water Productivity and Influencing Factors in the Yellow River Basin

#### 3.3.1. Correlation Characteristics between Water Productivity and Influencing Factors

**Figure 4:**
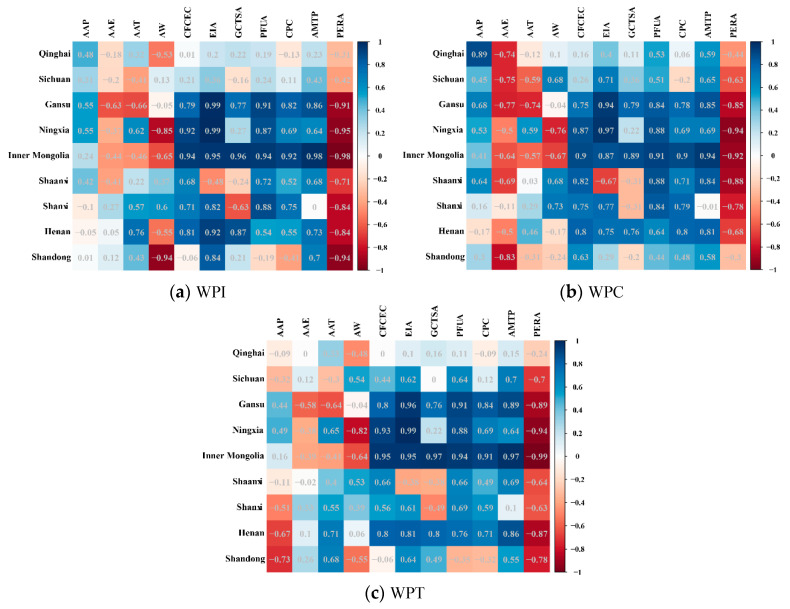
Correlation coefficients between water productivity and influencing factors. (**a**) Correlation coefficient between the WPI of the provinces (autonomous regions) and the influencing factors. (**b**) Correlation coefficient between the WPC of the provinces (autonomous regions) and the influencing factors. (**c**) Correlation coefficient between the WPT of the provinces (autonomous regions) and the influencing factors. The depth of the color indicates the size of the correlation coefficient. The lighter the color, the smaller the correlation coefficient, and the darker the color, the larger the correlation coefficient.

In Figure 4a, the correlation between WPI and the influencing factors was relatively small in the western Yellow River Basin (such as Qinghai and Sichuan). WPI showed a significant positive correlation with the consumption of chemical fertilizer with a 100% effective component (CFCEC), effective irrigated area (EIA), total sown area of grain crops (GCTSA), plastic film used for agriculture (PFUA), consumption of chemical pesticides (CPC), and total power of agricultural machinery (AMTP) in the northern Yellow River Basin (such as Inner Mongolia), and the correlation coefficients were all greater than 0.9; on the other hand, the WPI showed a significant negative correlation with the persons engaged in rural areas (PERA), with a correlation coefficient of −0.98. The WPI showed a significant positive correlation with CFCEC, EIA, and GCTSA in the eastern Yellow River Basin (such as Henan), with correlation coefficients of 0.81, 0.92, and 0.87, respectively, and showed a significant negative correlation between PERA and the WPI with a correlation coefficient of −0.84.

In Figure 4b, there was a significant positive correlation (0.89) between the average annual precipitation (AAP) and the WPC in the western Yellow River Basin (such as Qinghai). The WPC showed a significant positive correlation with CFCEC (0.90), EIA (0.87), GCTSA (0.89), PFUA (0.91), CPC (0.90), and AMTP (0.94) in the northern Yellow River Basin (such as Inner Mongolia), and the WPC showed a significant negative correlation with PERA (−0.92). In the eastern Yellow River Basin, the WPC showed a significant positive correlation with PFUA (0.84) in Shanxi, the AMTP had a significant positive correlation with the WPC (0.81) in Henan, and the average annual evaporation (AAE) had a significant negative correlation with the WPC (−0.83) in Shandong.

In Figure 4c, the correlation between WPT and the influencing factors was relatively small in the western (such as Qinghai and Sichuan) and eastern (such as Shanxi and Shandong) Yellow River Basin. In the northern Yellow River Basin, the WPT showed a significant positive correlation with CFCEC, EIA, GCTSA, PFUA, CPC, and AMTP in Inner Mongolia, with correlation coefficients of 0.95, 0.95, 0.97, 0.94, 0.91, and 0.97, respectively; the WPT showed a significant negative correlation with PERA in Inner Mongolia, with a correlation coefficient of −0.99. There was a significant positive correlation between WPT and CFCEC, EIA, and AMTP in the eastern (such as Henan) Yellow River Basin, with correlation coefficients of 0.81, 0.81, and 0.86, respectively, and there was a significant negative correlation between PERA and the WPT, with a correlation coefficient of −0.87.

On the whole, in the Yellow River Basin the water productivity had a significant positive correlation with CFCEC, EIA, PFUA, and AMTP, while it had a significant negative correlation with PERA.

#### 3.3.2. Grey Correlation and Sensitivity between Water Productivity and Influencing Factors

**Figure 5:**
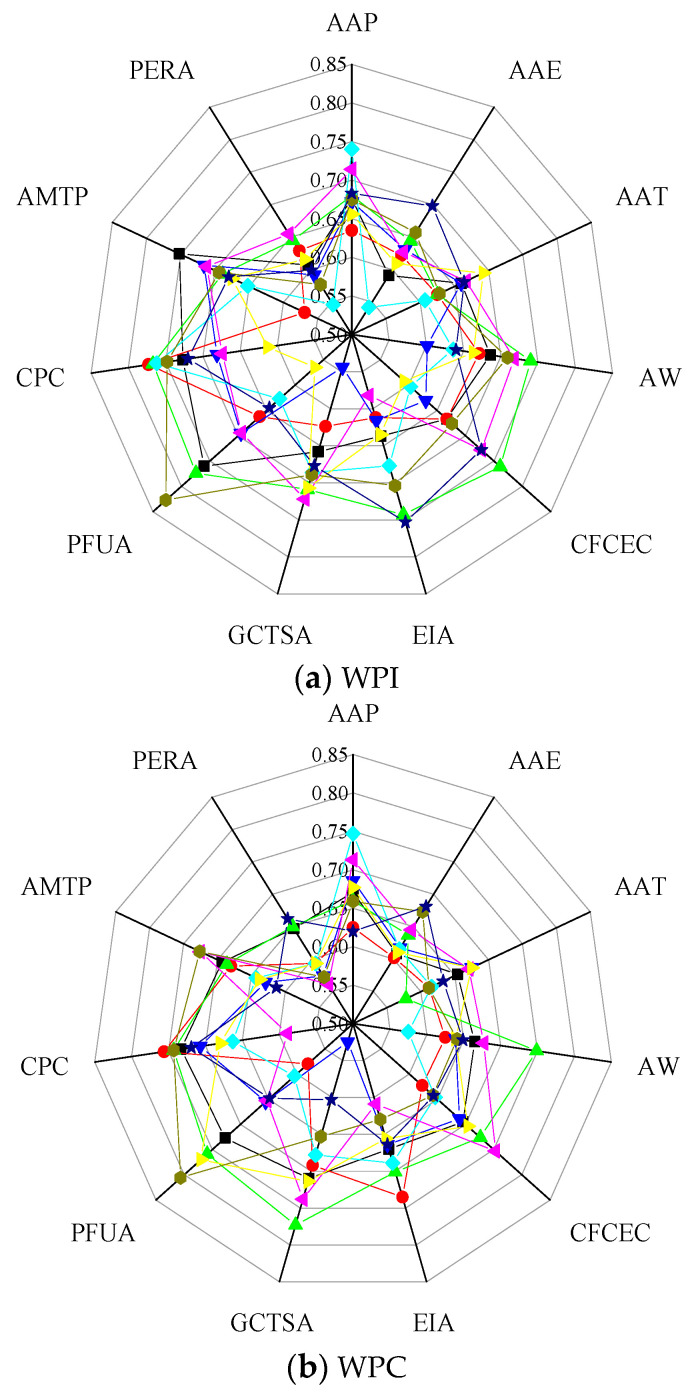

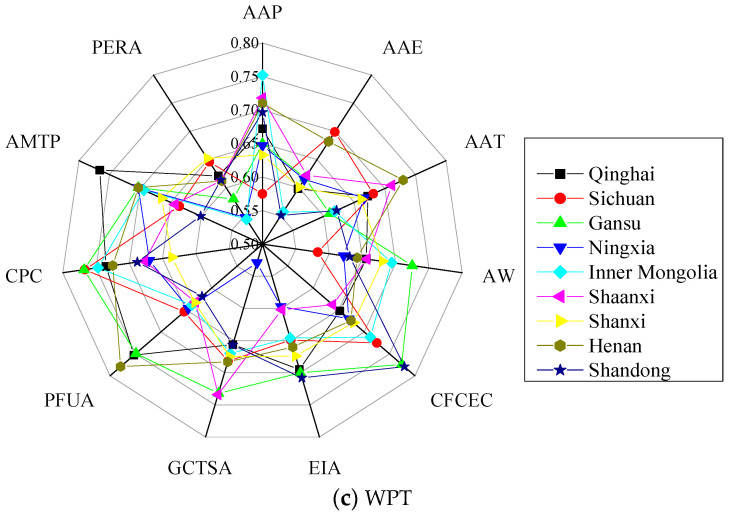
The grey correlation degree of the influencing factors with regard to water productivity. (**a**) Grey correlation degree of the influencing factors to WPI of the provinces (autonomous regions). (**b**) Grey correlation degree of the influencing factors with regard to WPC of the provinces (autonomous regions). (**c**) Grey correlation degree of the influencing factors with regard to WPT of the provinces (autonomous regions).

**Figure 6:**
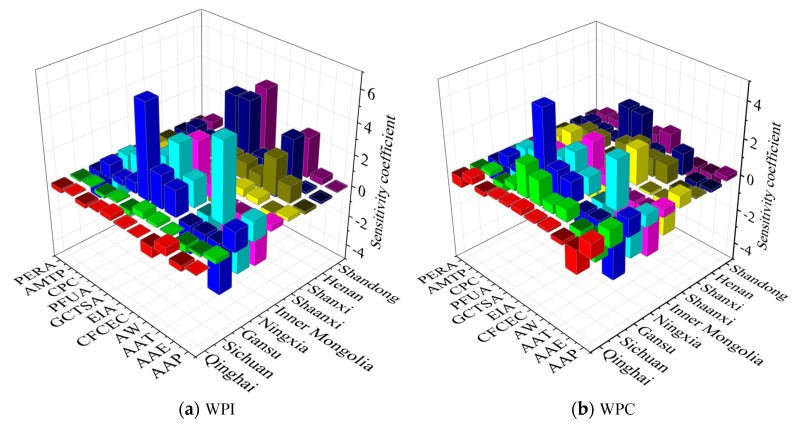

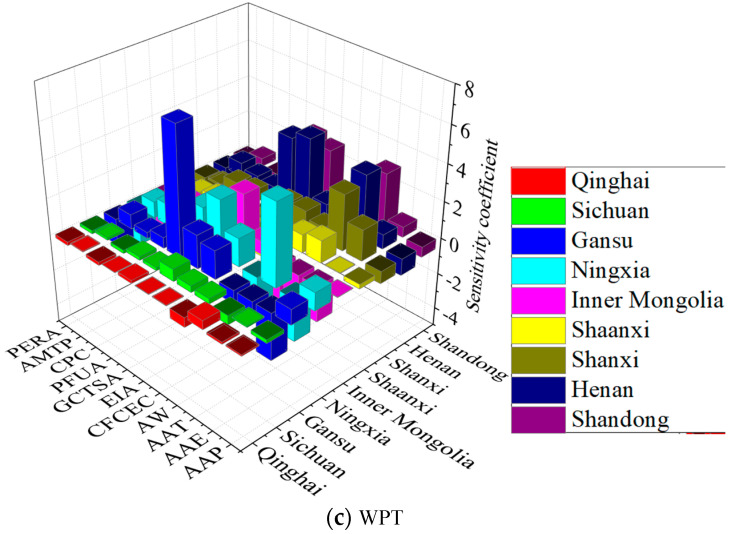
The sensitivity coefficient of the influencing factors with regard to water productivity. (**a**) Sensitivity coefficient of the influencing factors with regard to WPI of the provinces (autonomous regions). (**b**) Sensitivity coefficient of the influencing factors with regard to WPC of the provinces (autonomous regions). (**c**) Sensitivity coefficient of the influencing factors with regard to WPT of the provinces (autonomous regions).

The grey correlation and sensitivity between water productivity and the influencing factors are shown in Figure 5 and Figure 6, respectively. In Figure 5a and Figure 6a, the grey correlation degrees between WPI and PFUA, CFC, and AMTP in Qinghai were relatively larger, i.e., 0.760, 0.727, and 0.752, respectively; the grey correlation degrees of AAP and CFC with the WPI in Inner Mongolia were 0.740 and 0.762, respectively; and the maximum sensitivity coefficients of AW and WPI in Inner Mongolia were both −4.315, indicating that WPI decreases with increasing AW. The grey correlation degrees in Gansu (between WPI and AW, CFCEC, EIA, GCTSA, PFUA, and CFC) and Ningxia (between WPI and AMTP) were relatively larger, being all greater than 0.7; however, the maximum sensitivity coefficients of AAT and GCTSA for WPI in Ningxia and Gansu were 5.067 and 6.073, respectively, which indicates that the WPI increased with the increase in AAT and GCTSA. The grey correlation degree between CFC and WPI in Sichuan was 0.773, between GCTSA and WPI in Shanxi was 0.707, and between WPI and AAP, AW, CFCEC, GCTSA, and AMTP in Shaanxi was 0.714, 0.718, 0.728, 0.722, and 0.712, respectively; on the other hand, the maximum sensitivity coefficients of GCTSA and WPI in Shaanxi and Shanxi were −1.015 and −2.743, respectively, indicating that WPI decreased with the increase in GCTSA. The grey correlation degrees of AW, EIA, PFUA, and CFC with WPI in Henan were 0.709, 0.704, 0.827, and 0.748, respectively, whereas for CFCEC, EIA, and CFC with WPI in Shandong, the grey correlation degrees were 0.728, 0.753, and 0.720. The maximum sensitivity coefficients of EIA and WPI in Henan and Shandong were 4.125 and 4.371, respectively, which indicates that WPI in Henan and Shandong increased with the increase in EIA.

According to Figure 5b and Figure 6b, the grey correlation degrees were relatively larger between WPC and GCTSA (0.710), PFUA (0.727), and CFC (0.733) in Qinghai; between AAP and WPC (0.747) in Inner Mongolia; between CFC and WPC (0.719) in Shandong; between WPC and AAP (0.713), CFCEC (0.753), GCTSA (0.738), and AMTP (0.725) in Shaanxi; and between WPC and CFCEC (0.704), GCTSA (0.714), and PFUA (0.770) in Shanxi. The maximum sensitivity coefficients were relatively higher between AEE and WPC (−1.315) in Qinghai; between AW and WPC (−2.888) in Inner Mongolia; between EIA and WPC (−3.766) in Shaanxi; and between GCTSA and WPC in Shanxi (1.755) and Shandong (−1.552), indicating that WPC decreases with the increase in AEE, AW, EIA, and GCTSA. The grey correlation degrees were relatively larger between WPC and EIA (0.735) and CFC (0.756) in Sichuan; between WPC and AW (0.749), CFCEC (0.726), EIA (0.701), GCTSA (0.773), PFUA (0.759), and CFC (0.743) in Gansu; between CFC and WPC (0.707) in Ningxia; and between WPC and PFUA (0.806), CFC (0.743), and AMTP (0.726) in Henan. The maximum sensitivity coefficients were between AAT and WPC (2.710) in Ningxia, and between GCTSA and WPC in Sichuan (1.780), Gansu (4.330), and Henan (2.294), which indicates that WPC increased with the increase in AAT and GCTSA.

In Figure 5c and Figure 6c, the grey correlation between AMTP and WPT in Ningxia was relatively larger (0.703), and those of AAP, AAT, PFUA, CFC, and AMTP with WPT in Henan were 0.710, 0.730, 0.779, 0.725, and 0.703, respectively. The grey correlation of CFCEC and CFC with WPT in Sichuan was 0.725 and 0.767; that of AW, CFCEC, EIA, GCTSA, PFUA, CFC, and AMTP with WPT in Gansu was 0.725, 0.774, 0.700, 0.732, 0.749, 0.768, and 0.703, respectively; and that of CFCEC and EIA with WPT in Shandong was 0.779 and 0.708, respectively. The maximum sensitivity coefficients of AAT with WPT in Ningxia and Shanxi, EIA with WPT in Sichuan and Henan, and GCTSA with WPT in Gansu and Shandong were 4.472 and 3.015, 0.657 and 3.951, and 6.941 and 3.069, respectively, which indicates that WPT increased with the increase in AAT, AW, and GCTSA. The grey correlation degrees of PFUA, CFC, and AMTP with WPT in Qinghai were 0.753, 0.735, and 0.766, respectively; those of AAP, CFCEC, and CFC with WPT in Inner Mongolia were 0.752, 0.712, and 0.747; and those of AAP, AAT, and GCTSA with WPT in Shaanxi were relatively larger, being 0.718, 0.711, and 0.734, respectively. The maximum sensitivity coefficients of AW with WPT in Qinghai and Inner Mongolia were −0.516 and −3.692, respectively, and that of EIA with WPT in Shaanxi was −1.457, indicating that WPT decreased with the increase in AW and EIA.

In general, there was a large grey correlation degree between the water productivity of AAP, CFCEC, PFUA, CFC, and AMTP in the Yellow River Basin, but their sensitivity was relatively small. The sensitivity of water productivity to AAE, AAT, AW, EIA, GCTSA, and PERA was relatively high in the Yellow River Basin.

#### 3.3.3. The Relative Contribution Rate of the Influencing Factors to Water Productivity

**Figure 7:**
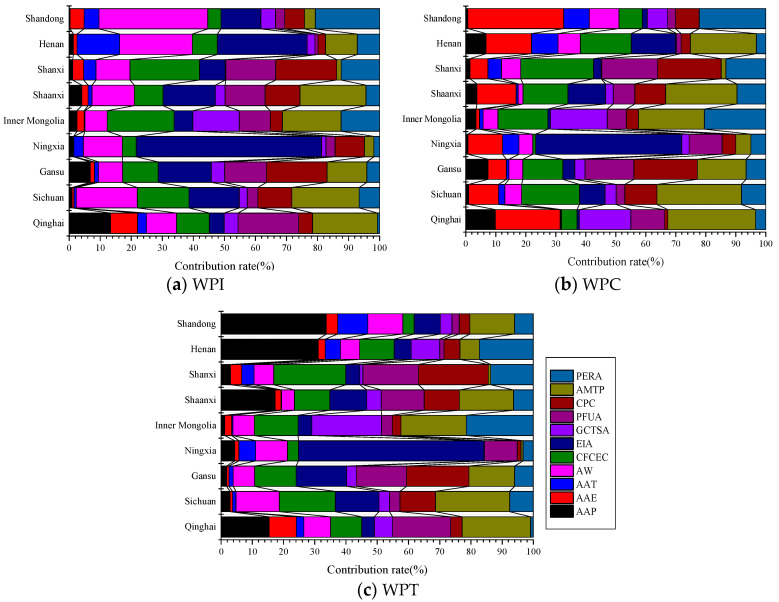
The relative contribution rate of the influencing factors to water productivity. (**a**) Relative contribution rate of the influencing factors to WPI of the provinces (autonomous regions). (**b**) Relative contribution rate of the influencing factors to WPC of the provinces (autonomous regions). (**c**) Relative contribution rate of the influencing factors to WPT of the provinces (autonomous regions).

The multiple linear regression equations between water productivity (WPI, WPC, and WPT) and the influencing factors were established according to Formula (11), and the relative contribution rates of each influencing factor to water productivity were calculated using Formula (12), as shown in Figure 7.

According to Figure 7a, the relative contribution rate of AMTP to WPI in Qinghai, Sichuan, and Shaanxi was the largest, being 20.86%, 21.69%, and 21.25%, respectively. The maximum relative contribution rate of CPC to WPI in Gansu was 19.50%, and the relative contribution rate of EIA to WPI in Ningxia and Henan was the largest, being 59.69% and 28.94%, respectively. The maximum relative contribution rates of CFCEC to WPI in Inner Mongolia and Shanxi were 21.55% and 22.43%, respectively, and the maximum relative contribution rate of AW to WPI in Shandong was 35.05%. According to Figure 7b, the relative contribution rate of AMTP to WPC in Qinghai, Sichuan, Inner Mongolia, Shaanxi, and Henan was the largest, being 29.25%, 28.19%, 22.01%, 23.79%, and 22.02%, respectively. The maximum relative contribution rate of CPC to WPC in Gansu was 21.17%, and the relative contribution rate of EIA to WPC in Ningxia was the largest, being 48.74%. The maximum relative contribution rate of CFCEC to WPC in Shanxi was 24.22%, and the relative maximum contribution rate of AAE to WPC in Shandong was 31.90%. According to Figure 7c, the relative contribution rates of AMTP to WPT in Qinghai and Sichuan was the largest, which were 21.91% and 23.74%, respectively. The maximum relative contribution rate of CPC to WPT in Gansu was 19.99%, and the relative contribution rate of GCTSA to WPT in Inner Mongolia was the largest, being 22.32%. The maximum relative contribution rate of EIA to WPT in Ningxia was 59.43%, and the maximum relative contribution rate of CFCEC to WPT in Shanxi was 22.98%. The relative contribution rate of AAP to WPT in Shaanxi, Shandong, and Henan was the largest: 17.35%, 31.13%, and 33.70%, respectively.

Overall, although the contribution of the influencing factors to the water productivity had certain differences in different provinces, the major contributing factors to WPI were EIA, AW, AMTP, CFCEC, and CPC in the Yellow River Basin, in which the average relative contribution rates were 18.98%, 15.55%, 12.64%, 12.06%, and 9.77%, respectively. The AMTP, CFCEC, AAE, EIA, and PERA contributed significantly to WPC in the Yellow River Basin, and their average relative contribution rates were 16.46%, 13.25%, 12.94%, 10.49%, and 10.19%, respectively. The EIA, AMTP, AAP, CFCEC, and PFUA contributed significantly to WPT in the Yellow River Basin, and the average relative contribution rates were 14.26%, 13.38%, 12.30%, 10.49%, and 9.69%, respectively.

## 4. Discussion

Water productivity measures how the crop output benefits from a certain water resource input, and it is an important parameter to evaluate the management level of agricultural irrigation water and the efficiency of water-saving developments. Comparing the temporal and spatial differentiation characteristics of water productivity is an important basis for evaluating the developmental effects of water-saving irrigation. At present, most studies focus on the comparative analysis of small-scale water productivity in the field and medium-scale water productivity in irrigated areas to explore the scale effect of water productivity. One related study showed that the yield of seed cotton increased with the increase in the irrigation amount, whereas the water productivity decreased with the increase in the irrigation amount [48]. Another found that the water consumption of cotton under mulch drip irrigation in Xinjiang during the whole growth period should be 345~380 mm when the water productivity is high [49]. A study demonstrated that the water productivity increased with the increase in scale due to the reuse of regressed water through the field experiments, remote sensing, and hydrological models in the Zhanghe Irrigation District, Hubei Province, China [50,51,52,53]. Another study showed that the net irrigated water productivity showed that the water productivity decreased significantly with the increase in scale in Shijing Irrigation District by testing at different scales [54]. As it was difficult to accurately obtain the measured data of agricultural production and agricultural water consumption through experimental methods on a large spatial scale, one related study showed that the water productivity of the provinces in and around Huang-Huai-Hai Plain was high, whereas provinces in the northeast, south of the Yangtze River, and in the northwest of China showed lower water use efficiency [55]. Another study showed that the water productivity of winter wheat in the Haihe River Basin showed a steady increasing trend between years [26]. One study demonstrated that the average annual comprehensive water productivity in the northern and coastal areas of Liaoning Province was higher, whereas that in the central area was smaller [56]. Another study showed that the spatial distribution of water productivity in Xi’an was similar to the spatial distribution of water productivity in different years [35]. In this study, the water productivity is relatively small in the western region and relatively large in the eastern region in the Yellow River Basin, which indicates that water productivity is closely related to regional water resource conditions.

Water productivity is closely related to regional climate conditions, water resource endowment, crop types, and other natural factors, and is also affected by the planting structure, food types, agricultural production and management mode, farmland water conservancy facilities, and the socioeconomic development degree [57]. The results showed that the AAT, CFCEC, EIA, GCTSA, and AMTP were the main factors affecting WPC in Xi’an [35], which was consistent with the main influencing factors (AAE, CFCEC, EIA, CPC, and AMTP) affecting the WPC in Shaanxi in this study. The main factors affecting the water productivity of corn in an oasis irrigated area included five controllable factors: the PFUA, CFCEC, the seeds for production, the labor input, and the CPC [58]. Li et al. showed that agronomic measures (AW, CFCEC, PFUA, and CPC), daily average temperature, and solar radiation were the main factors affecting WPI [59]. Another study showed that the sowing area, yield per unit, and irrigation water consumption were the key factors affecting the WPI of corn in Liaoning Province [60]. As for the sensitivity analysis, relevant studies showed that the sensitivity of wheat water productivity to relative humidity and wind speed was higher at the field and regional scale, but the sensitivity to sunshine duration was relatively lower; additionally, it was found that the sensitivity of crop water productivity to irrigation efficiency was higher, but the sensitivity to fertilizer consumption was lower [61]. The sensitivity of irrigation water was higher than that of the fertilization amount when the hydrological age was changed from flood year to flat year, and then to low-flow year in North China [62]. In this study, although there are some differences in the sensitivity of water productivity to influencing factors among provinces in the Yellow River Basin, the sensitivity of water productivity to AAE, AAT, AW, EIA, GCTSA, and PERA was relatively high.

Water productivity is an important index of the efficient utilization of agricultural water, and improving water productivity is of great significance for the rational utilization of water resources and ensuring food security. In order to improve the water productivity of the study area, which is necessary to improve the water-saving irrigation mode, it is necessary to abandon the traditional extensive use of water resources, such as through flood irrigation and flooded irrigation. The spatial mismatch between grain production and population distribution results in a large amount of grain transport between provinces in the Yellow River Basin, among which Henan, Shandong, and Inner Mongolia are the main grain export regions. The related studies showed that if the water-intensive agricultural products are transferred from regions with high water resource utilization efficiency to regions with low water resource utilization efficiency, the water resource pressure can be relieved and water resources can be saved in the Yellow River Basin [63,64]. The eastern region of the Yellow River Basin had obvious comparative advantages in the grain water production efficiency, which was conducive to the conservation of water resources compared with the grain production in other provinces in the Yellow River Basin. In order to increase the availability of water for grain production in the Yellow River Basin, modern water-saving agricultural practices should be further developed to reduce the ineffective loss of water resources in the process of agricultural production, and to increase the supply of water that is transregionally transferred for agriculture and ecology [65]. In addition, due to the limitation of climate conditions, in the case that it is difficult to increase the multiple cropping index, expanding the irrigation area or transferring water resources to industries or services with added production value is an effective way to improve the efficiency of water resource utilization.

## 5. Conclusions

The results showed that the minimum mean values of WPI, WPC, and WPT were 0.22, 0.35, and 0.18 kg/m^3^, respectively, in the western Yellow River Basin (such as Qinghai). In the eastern Yellow River Basin, the maximum mean value of WPI was 2.11 kg/m^3^ in Henan, and the maximum mean values of WPC and WPT were 0.71 and 0.61 kg/m^3^, respectively, in Shandong. The water productivity displayed a certain spatial clustering feature in the Yellow River Basin, such as the WPI of Henan showing H-H aggregation in 2005, the WPI of Shanxi showing H-H aggregation in 2015, and the WPI of Gansu showing L-L aggregation in 2005, 2010, and 2015; the concentration of WPT in all provinces in 2005, 2010, and 2015 was not significant.

The water productivity had a significant positive correlation with the consumption of chemical fertilizer with a 100% effective component (CFCEC), effective irrigated area (EIA), plastic film used for agriculture (PFUA), and total power of agricultural machinery (AMTP), whereas it had a significant negative correlation with the persons engaged in rural areas (PERA). The grey correlation degrees between the water productivity and the average annual precipitation (AAP), CFCEC, PFUA, consumption of chemical pesticides (CFC), and AMTP were relatively larger, but their sensitivity was relatively small; furthermore, the sensitivity of water productivity to average annual evaporation (AAE), annual average temperature (AAT), agricultural water (AW), EIA, total sown area of grain crops (GCTSA), and PERA was relatively higher in the Yellow River Basin.

The main driving factors were the EIA, AW, AMTP, CFCEC, and CPC for WPI, for which the average relative contribution rates were 18.98%, 15.55%, 12.64%, 12.06%, and 9.77%, respectively; the main driving factors were AMTP, CFCEC, AAE, EIA and PERA for WPC, and their average relative contribution rates were 16.46%, 13.25%, 12.94%, 10.49%, and 10.19%, respectively; and the main driving factors were the EIA, AMTP, AAP, CFCEC, and PFUA for WPT, and the average relative contribution rates were 14.26%, 13.38%, 12.30%, 10.49%, and 9.69%, respectively.

## Figures and Tables

**Table 1:** Basic information of the indicators.

Number	Name	Abbreviation	Unit
1	irrigation water productivity	WPI	kg/m^3^
2	crop water productivity	WPC	kg/m^3^
3	total inflow water productivity	WPT	kg/m^3^
4	annual average precipitation	AAP	mm
5	annual average evaporation	AAE	mm
6	annual average temperature	AAT	°C
7	agricultural water	AW	10^8^ m^3^
8	consumption of chemical fertilizer with a 100% effective component	CFCEC	10,000 tons
9	effective irrigated area	EIA	1000 hectares
10	total sown area of grain crops	GCTSA	1000 hectares
11	plastic film used for agriculture	PFUA	ton
12	consumption of chemical pesticides	CPC	ton
13	total power of agricultural machinery	AMTP	10,000 kw
14	persons engaged in rural areas	PERA	10,000 person

**Table 2:** The statistical characteristics of water productivity in the Yellow River Basin from 2000 to 2019.

Indicators	Statistics	Qinghai	Sichuan	Gansu	Ningxia	Inner Mongolia	Shaanxi	Shanxi	Henan	Shandong
WPI	Maximum value (kg/m^3^)	0.27	1.14	0.75	0.53	1.23	1.00	1.54	2.69	2.48
	Minimum value (kg/m^3^)	0.17	0.89	0.27	0.15	0.43	0.72	0.77	1.39	1.34
	Mean value (kg/m^3^)	0.22	1.00	0.46	0.32	0.86	0.83	1.22	2.11	1.93
	Standard deviation	0.02	0.06	0.13	0.11	0.25	0.06	0.20	0.36	0.32
	CV/%	10.31	5.69	28.48	34.68	28.88	7.26	16.25	16.88	16.55
WPC	Maximum value (kg/m^3^)	0.42	0.61	0.52	0.59	0.52	0.55	0.54	0.73	0.85
	Minimum value (kg/m^3^)	0.25	0.40	0.24	0.30	0.27	0.34	0.30	0.50	0.53
	Mean value (kg/m^3^)	0.35	0.50	0.36	0.43	0.40	0.45	0.45	0.65	0.71
	Standard deviation	0.04	0.06	0.07	0.08	0.08	0.07	0.07	0.07	0.08
	CV/%	11.33	11.22	20.28	19.50	20.38	14.72	15.61	10.77	11.20
WPT	Maximum value (kg/m^3^)	0.22	0.60	0.48	0.41	0.75	0.37	0.59	0.78	0.79
	Minimum value (kg/m^3^)	0.14	0.46	0.22	0.14	0.32	0.25	0.25	0.30	0.42
	Mean value (kg/m^3^)	0.18	0.52	0.34	0.26	0.54	0.32	0.44	0.54	0.61
	Standard deviation	0.02	0.03	0.08	0.08	0.13	0.03	0.08	0.10	0.08
	CV/%	9.15	5.83	23.09	29.80	24.90	9.41	18.28	19.07	13.88

**Table 3:** The Global Moran’s *I* coefficient of water productivity in the Yellow River Basin from 2000 to 2019.

Year	WPI		WPC		WPT	
	Moran’s *I*	*P*	Moran’s *I*	*P*	Moran’s *I*	*P*
2000	0.431	0.472	0.243	0.330	0.147	0.558
2001	0.410	0.559	0.172	0.104	0.211	0.649
2002	0.418	0.475	0.358	0.269	0.178	0.649
2003	0.469	0.649	0.336	0.398	0.072	0.845
2004	0.491	0.649	0.254	0.398	0.276	0.745
2005	0.456	0.649	0.169	0.051	0.326	0.948
2006	0.504	0.649	0.473	0.559	0.416	0.845
2007	0.463	0.649	0.377	0.269	0.322	0.745
2008	0.482	0.745	0.360	0.217	0.331	0.948
2009	0.471	0.649	0.271	0.217	0.224	0.948
2010	0.471	0.745	0.318	0.330	0.266	0.948
2011	0.461	0.745	0.464	0.559	0.132	0.845
2012	0.505	0.845	0.396	0.398	0.296	0.845
2013	0.531	0.845	0.455	0.845	0.253	0.948
2014	0.493	0.845	0.378	0.398	0.262	0.948
2015	0.491	0.845	0.396	0.745	0.244	0.948
2016	0.501	0.845	0.478	0.474	0.170	0.948
2017	0.493	0.845	0.400	0.398	0.171	0.948
2018	0.498	0.845	0.292	0.329	0.263	0.948
2019	0.485	0.845	0.156	0.329	0.247	0.948

## Data Availability

All data are included in the manuscript. Additional information is available upon request from the corresponding authors.
